# Epstein-Barr virus meningitis mimicking tuberculous meningitis on neuroimaging

**DOI:** 10.1590/0037-8682-0254-2022

**Published:** 2022-09-19

**Authors:** Diogo Goulart Corrêa, Luis Alcides Quevedo Cañete, Luiz Celso Hygino da Cruz

**Affiliations:** 1 Clínica de Diagnóstico por Imagem/DASA, Departamento de Radiologia, Rio de Janeiro, RJ, Brasil.; 2 Universidade Federal Fluminense, Departamento de Radiologia, Niterói, RJ, Brasil.

**FIGURE 1:**
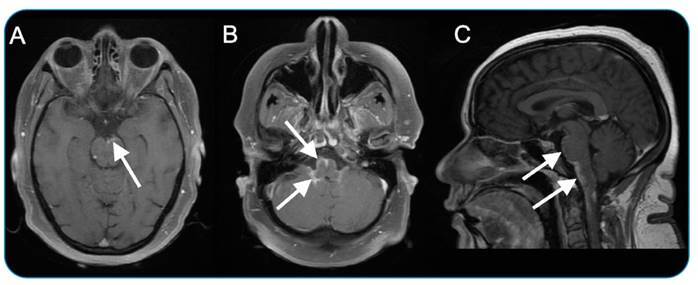
EBV meningitis. T1-weighted brain MRI with and without fat saturation after intravenous gadolinium injection (**axial, A and B; sagittal, C**) revealed abnormal leptomeningeal gadolinium enhancement in the basal cisterns, including the interpeduncular, pre-pontine, and pre-medullary cisterns, extending to the cervical spinal cord **(arrows)**.

A 44-year-old woman presented with a fever and cough for five days. After three days of symptoms, she presented with paresis of the four limbs, diplopia, vomiting, and dysphagia. Magnetic resonance imaging (MRI) of the brain showed leptomeningeal gadolinium enhancement in the basal cisterns (interpeduncular, pre-pontine, and premedullary) extending to the cervical spinal cord (Figure 1). The serology results for the human immunodeficiency virus were negative. Hemogram and computed tomography of the chest were normal. Analysis of the cerebrospinal fluid (CSF) revealed a pressure of 26 cm H_2_O, a glucose level of 35 mg/dL, a white blood cell count of 229/dL (93% lymphocytes), and a protein content of 107 mg/dL. The polymerase chain reaction of the CSF was positive for Epstein-Barr virus (EBV) and negative for herpes simplex virus, varicella-zoster virus, cytomegalovirus, enterovirus, and Mycobacterium tuberculosis. The venereal disease and fluorescent treponemal antibody test results were negative. The patient was diagnosed with EBV meningitis and treated with intravenous acyclovir and methylprednisolone.

Primary EBV infection commonly occurs via orofacial contamination and has a lifelong latency period. EBV meningitis may occur during primary infection but is usually due to reactivation from lymphoid follicles or migration of infected B lymphocytes following impairment of the cellular immune response^1^. EBV meningitis usually shows normal MRI findings of the brain but may also present with meningeal enhancement^2^. EBV meningoencephalitis typically manifests as lesions in the basal ganglia and thalamus.

Basal cisternal contrast enhancement occurs in tuberculous meningitis, neurosarcoidosis, leptomeningeal carcinomatosis, and fungal meningitis^3^. Considering EBV meningitis in the differential diagnosis of basal cisternal leptomeningeal contrast enhancement is important.
